# Supplementary material for: Structural Consequences of Deproteinating the 50S Ribosome
Source: Biomolecules. 2022 Oct 31;12(11):1605. doi: 10.3390/biom12111605 (PMC9687910; doi:10.3390/biom12111605)
Supplement: Supplementary file 1 [file biomolecules-12-01605-s001.zip › biomolecules-1973991-supplementary.pdf]

Supplemental material for:

Structural consequences of deproteinating the 50S ribosome

Daniel S. D. Larsson [a], Sandesh Kanchugal P [a#] & Maria Selmer [a*]

[a]Department of Cell and Molecular Biology, Uppsala University, Box 596, SE 751 24 Uppsala, Sweden

[#]Present address: MAX IV Laboratory, Fotongatan 2, SE 224 84 Lund, Sweden

[*]Address correspondence to Maria Selmer, maria.selmer@icm.uu.se.

Content:

Figure S1-S15

Table S1-S4

Supplementary references

Supplementary Figures

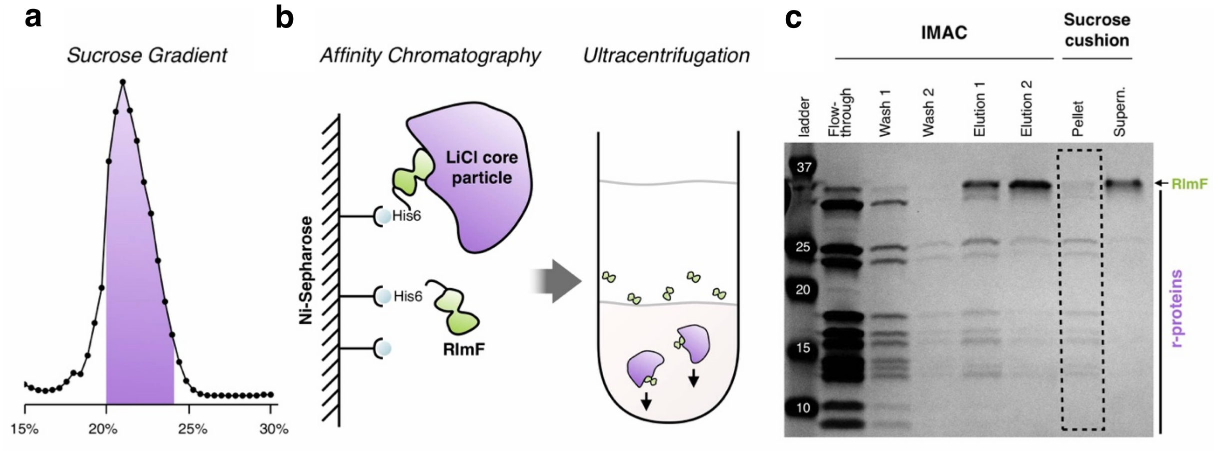

Figure S1. Sample preparation for cryo-EM imaging. (**a**) LiCl washed ribosomal were sedimented through a sucrose density gradient. Pooled fractions are shown in purple. (**b**) LiCl washed ribosomal LSUs (purple) with affinity for RlmF (green) were pulled down by the C-terminally hexa-histidine tagged enzyme in an IMAC step. Free enzyme was removed in the pelleting step through a sucrose cushion. (**c**) SDS-PAGE of fractions from the procedure. The molecular weight of His-tagged RlmF is 35.3 kDa and the largest LSU r-protein has a molecular weight around 30 kDa.

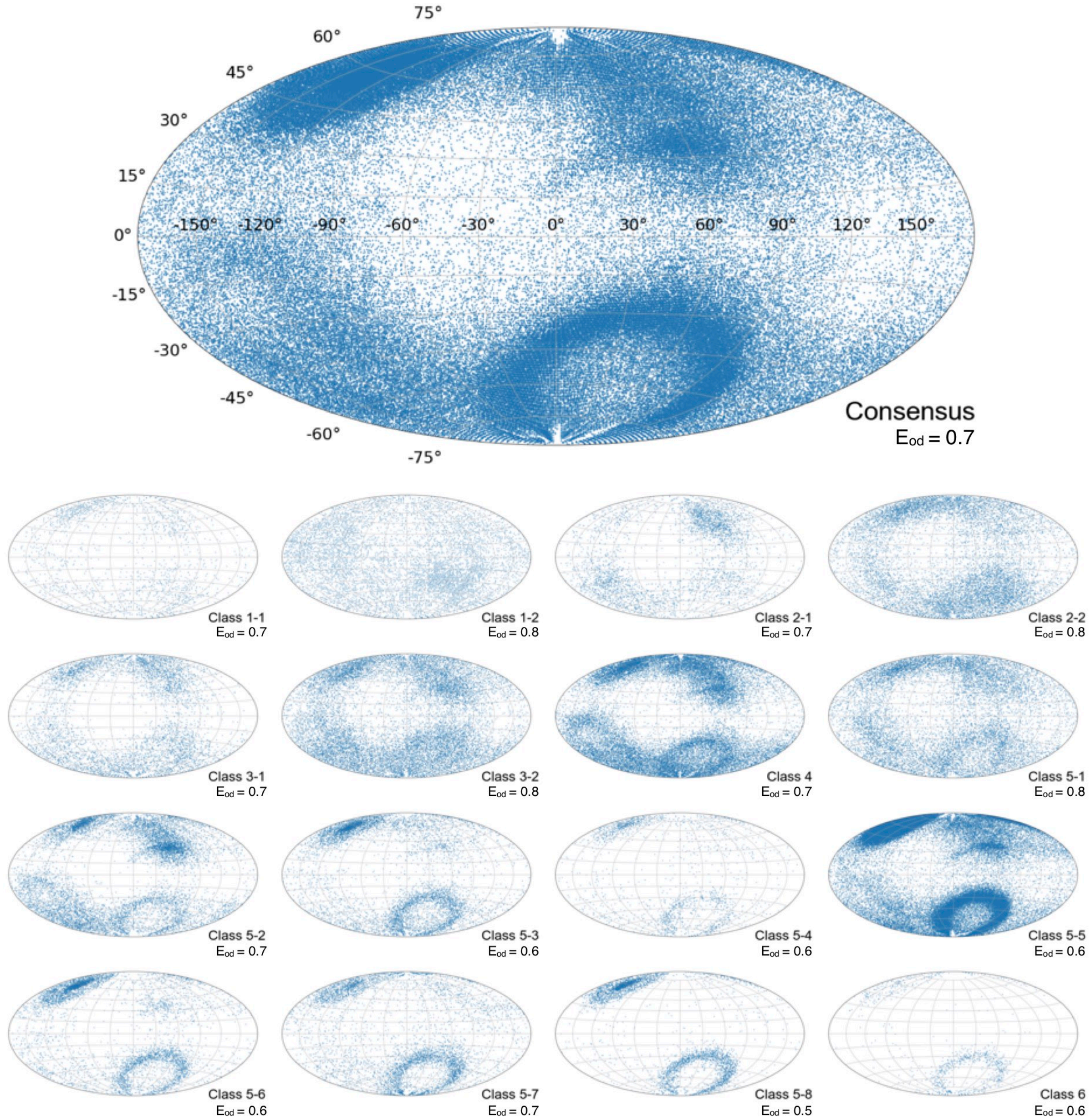

Figure S2. Euler angles from single-particle reconstructions plotted using the Hammer projection (area-preserving). The hot-spot at approximately -135°, +50° (most easily seen in class 5-6) represents the most preferred orientation in the non-tilted data set. The diffuse ring surrounding it comes mostly from particles in the 15°-tilted data set. The ring centered the almost exact opposite side, centered at +45, -55°, represents the preferred orientation in the 30°-titled data set. The 30°-tilted data was most likely collected with the grid upside-down in the microscope column relative to the other data collection session. Due to the projection transformation in TEM, the lower and upper hemispheres are redundant (*cf.* Friedel symmetry).

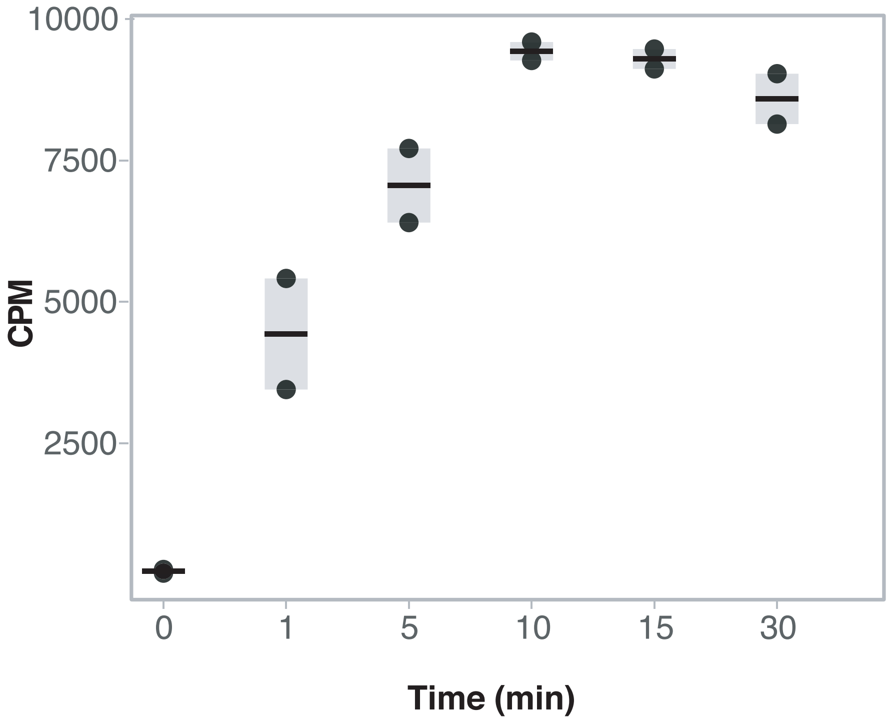

Figure S3. *In vitro* methylation assay. Time course measurement of methylation by RlmF where points represent technical duplicates for each time point and lines the mean value. The negative control without RlmF was measured to 223 +/- 32 cpm after 30 min.

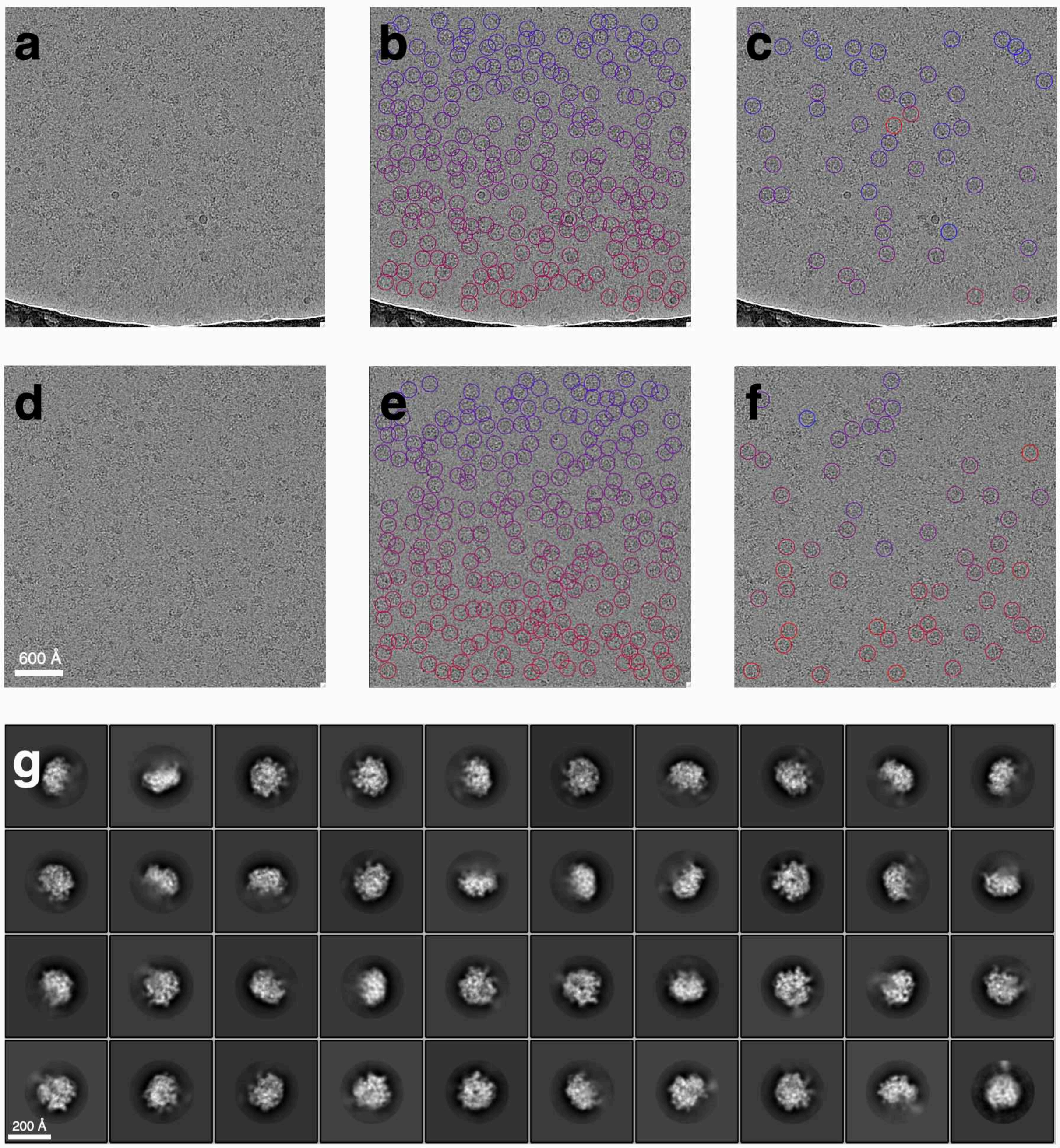

Figure S4. Examples of raw micrographs, particle picking and 2D classification. (**a-f**) Each row shows an example micrograph. Left column (**a,d**): Motion corrected micrographs low-pass filtered to 20 Å. Middle column (**b,e**): Initial particle locations as found using the Laplacian-of-Gaussian filtering method in RELION [1]. Right column (**c,f**): Particles retained in the final consensus reconstruction after multiple rounds of 2D and 3D classification to remove contaminations and unstructured particles. Color of rings represents the local defocus values, with values in the middle column estimated by gCTF [2] and in the right column further refinement using CtfRefine in RELION (the gradient is due to both micrographs being collected at 30° tilt of the sample stage). (**g**) Class averages after multiple rounds of reference-free 2D classification of particles. Classes show clear secondary structure features, but also significant degree of diffuse density in some views.

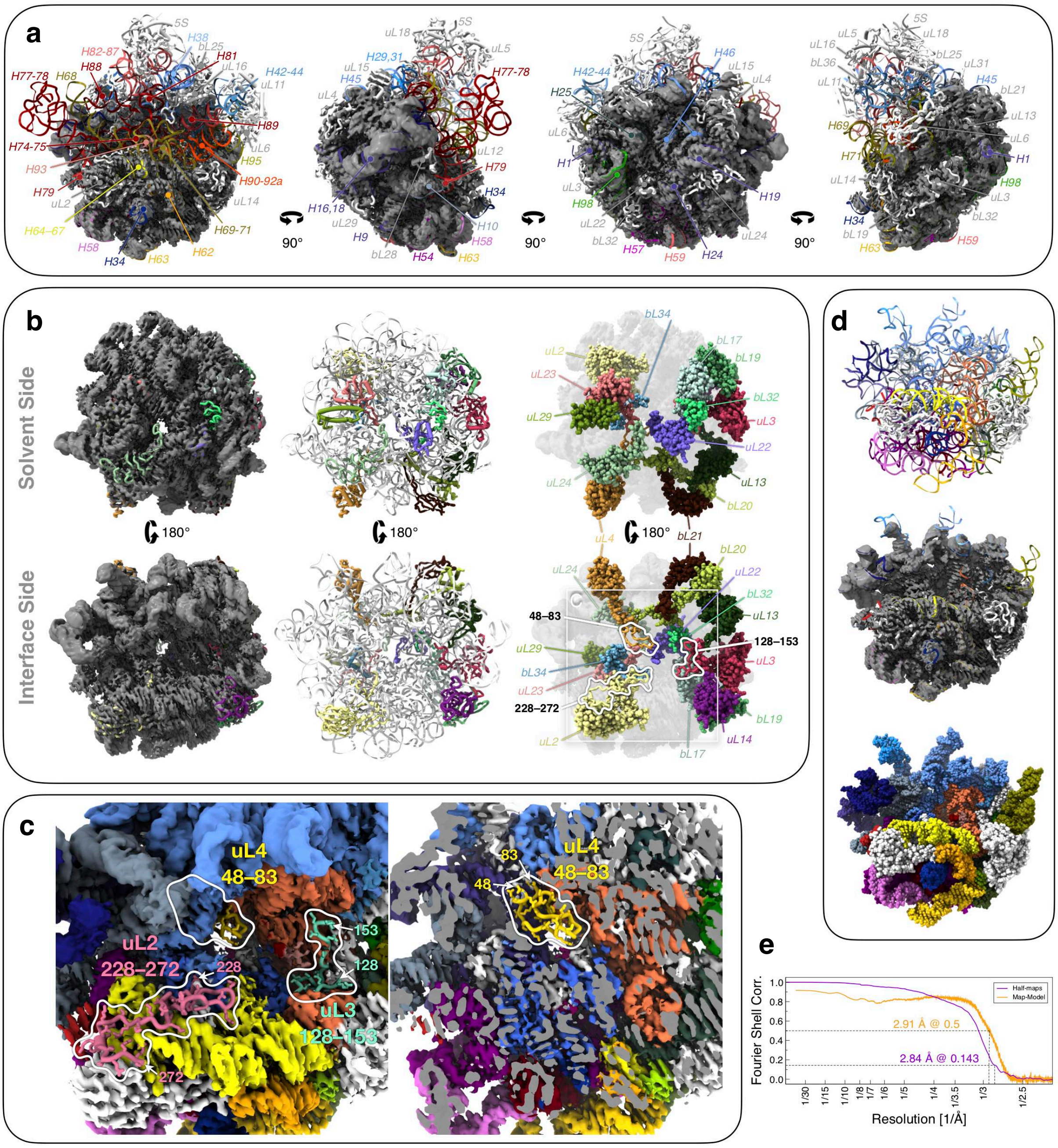

Figure S5. Related to Figure 2. Consensus reconstruction and model refinement. (**a**) Consensus reconstruction with an annotated rigid-body fitted model of mature 50S (PDB ID 4YBB) in four different orientations (same views as in Figures 2c). Model colored as in Figure 2b. (**b**) R-proteins in the consensus reconstruction, shown from the solvent side (top row) and the subunit interface side (bottom row). The left column shows the map of the consensus reconstruction and a model refined into it. Some proteins, such as uL14, uL24 and bL32 have weaker density, indicating partial occupancy or flexibility. The middle column shows just the refined model (RNA in white and r-proteins colored in unique colors). The right column shows the r-proteins as space-filling spheres. Disordered loops (labeled) are shown as cartoon (compare with (c)) and the map is shown in the background for reference. (**c**) Flexible loops in r-proteins uL2, uL3 and uL4. (Left) The consensus reconstruction together with modelled loops not supported by the density, as they are positioned in the mature particle. The field of view is indicated in (b). (Right) The same view as on the left side, but hiding the top part of the density to reveal the entire flexible part of protein uL4. The gray

surfaces indicate where density has been cut away. (**d**) Model refined in the consensus reconstruction, shown in the crown view (same view as Figure 2a) with (top) showing just the map (middle) showing both the map and the refined model as cartoon (colored as in Figure 2b) and (bottom) only the model, shown as space-filling spheres. (**e**) Fourier shell correlation between two independently reconstructed half-maps (purple) and between the full map and the refined model (orange).

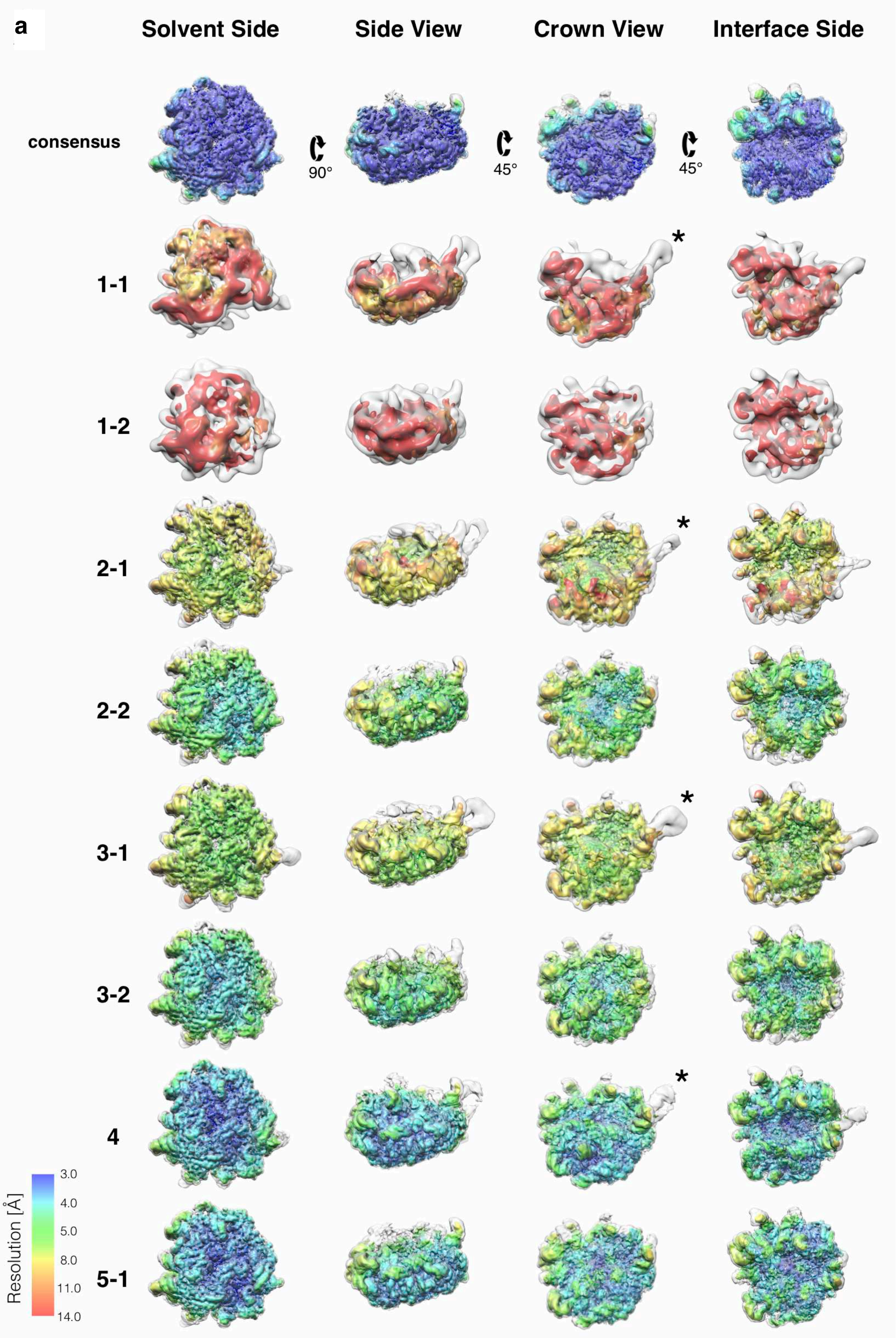
a
Solvent Side
Side View
Crown View
Interface Side
consensus
90°
45°
45°
1-1
1-2
2-1
2-2
3-1
3-2
4
5-1
*
Resolution [Å]
3.0
4.0
5.0
8.0
11.0
14.0

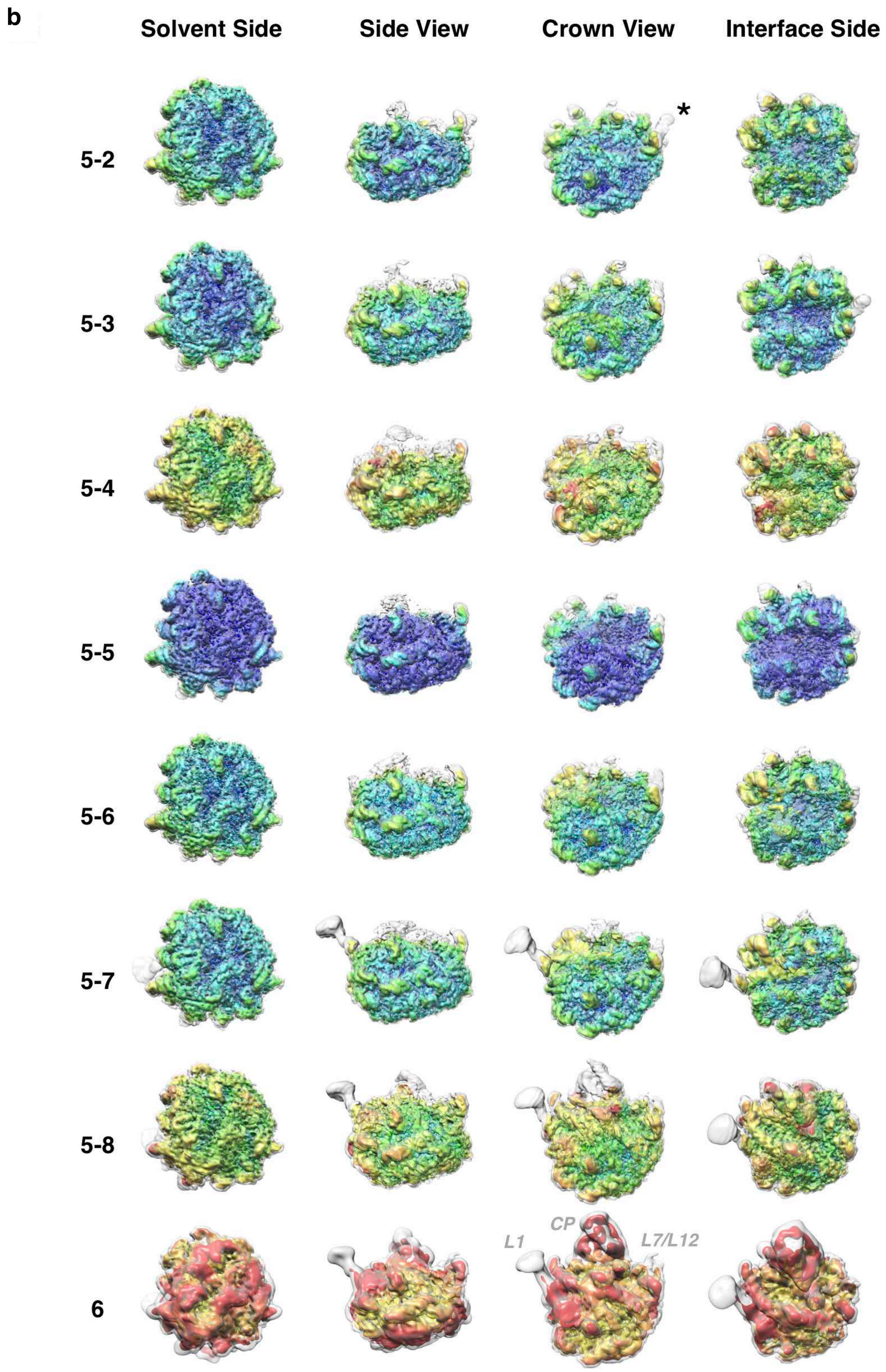
b
Solvent Side
Side View
Crown View
Interface Side
5-2
*
5-3
5-4
5-5
5-6
5-7
5-8
6
L1
CP
L7/L12

Figure S6. Non-redundant major and minor reconstruction classes. (**a–b**) The left-most column shows a view looking from the solvent side of the particle, approximately centered on the nascent-chain exit tunnel. The next column shows a view rotated 90° around the X-axis from the H63 side of the particle with the solvent side down and the subunit interface side up. The "Crown View" column is rotated 45° further with the central protuberance pointing up and the L1 protuberance to the left and the L7/L12 stalk to the right (*cf.* class 6). The right-most column shows the subunit interface side. Maps are locally low-pass filtered using RELION with B-factors as described in Table S2 and rendered as isosurfaces at map level 0.1 colored according to the estimated local resolution. A second, semi-transparent isosurface is also shown at a lower threshold (map level 0.05) to visualize flexible elements for maps that have additionally been globally low-pass filtered to 6 Å. Asterisks indicate loop-shaped non-native density close to the L7/L12 stalk base (see text).

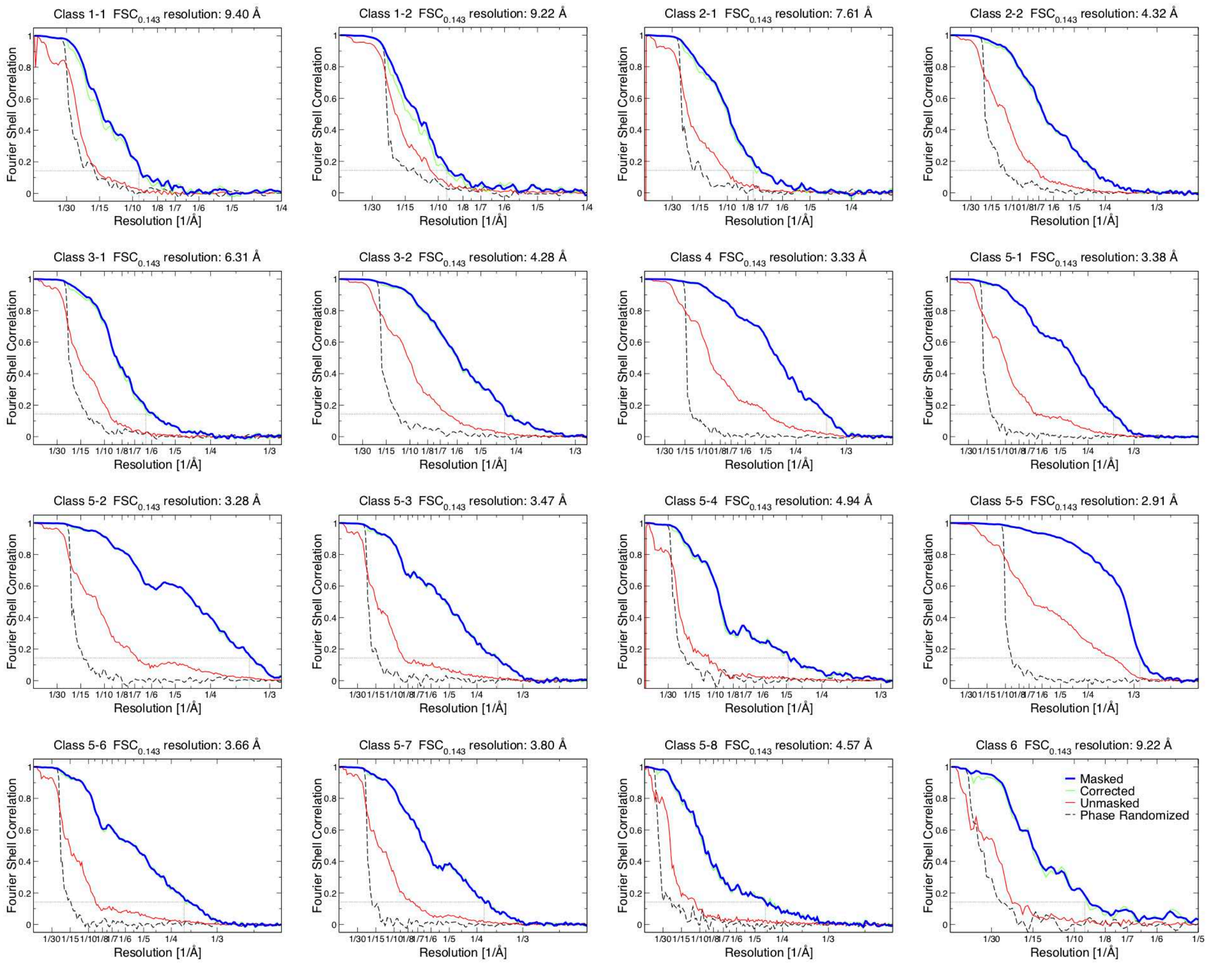

Figure S7. Fourier-shell correlation between independent half-sets during single-particle reconstructions of each class. The resolution where the curve crosses 0.143 is indicated with a dotted line.

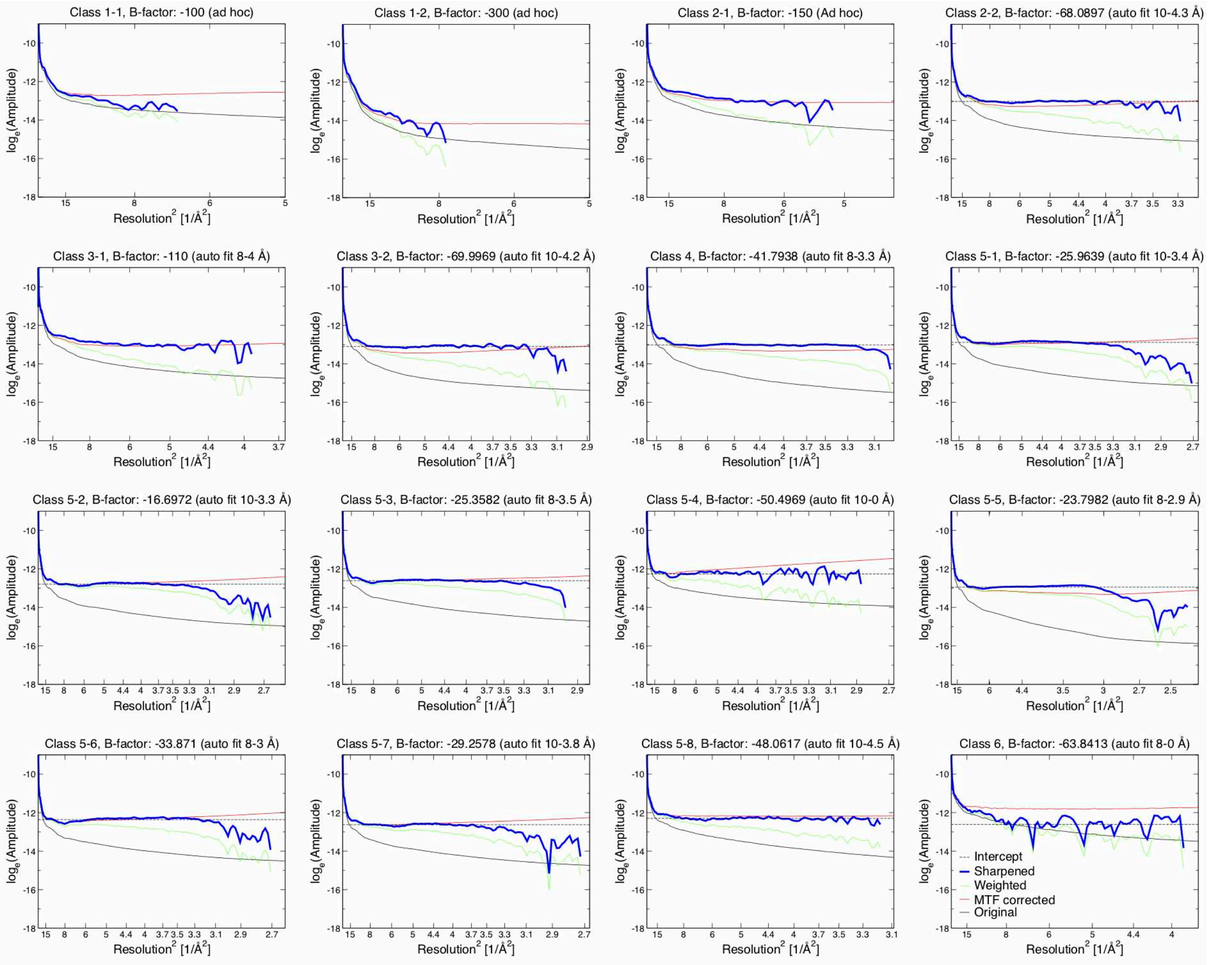

Figure S8. Guinier plots from the single-particle reconstructions of each class with linear curve fit at the approximately linear regime for B-factor sharpening.

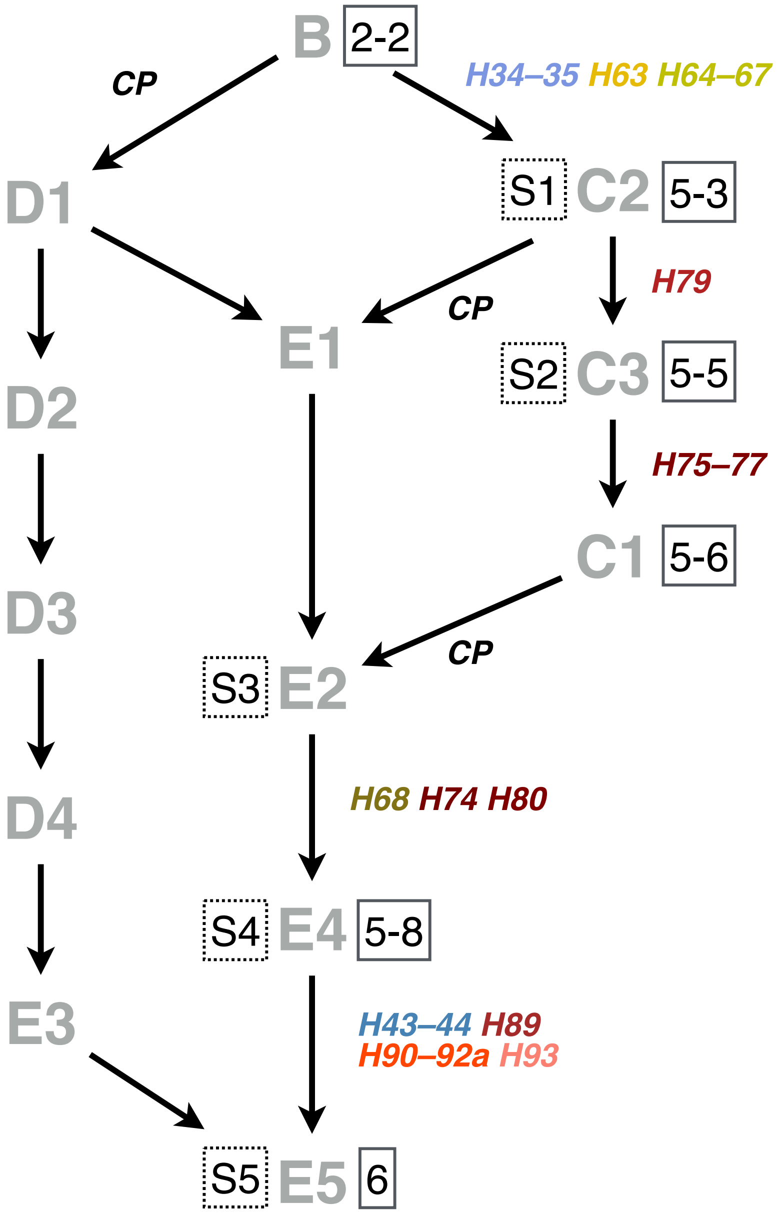

Figure S9. Comparison of 50S sub-particles formed during assembly and disassembly. Assembly pathways for the 50Ssubunit based on a bL17 depletion model [3] are shown with assembly intermediates in gray (b–e designations). Arrows connect nodes along the proposed paths, with folding/binding of important structural elements indicated (colors according to Figure 2b). Comparable particles isolated from *in vitro* reconstitution (S designations, dotted-line boxes) [4] and from LiCl washed ribosomes (this study, solid-line boxes). The LiCl washed ribosomes, as well as the *in vitro* reconstituted particles, are most similar to particles along the C/E pathway in the bL17 depletion model.

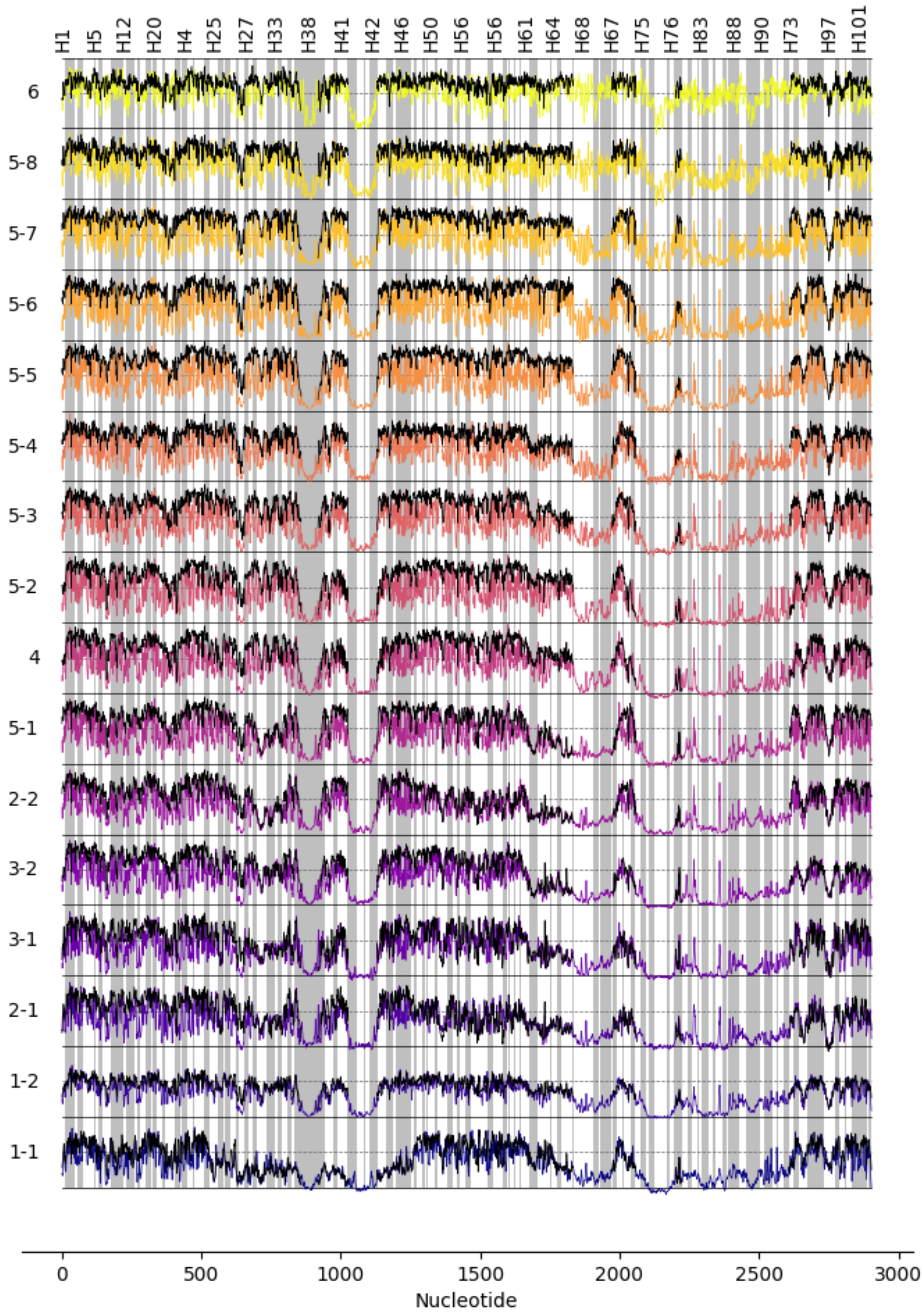

Figure S10. Correlation between map and model for the 23S rRNA, calculated as the average map values at heavy atom positions for each nucleotide in rigid-body docked atomic models. Colored lines are for a mature ribosome model (PDB ID 4YBB) and black lines are for a model refined against the consensus reconstruction. Vertical grey and white bars indicate secondary structure elements (selected helices labeled at the top). Dashed horizontal lines indicate a map value of 0.05 (threshold used for occupancy calculations in Figure S12).

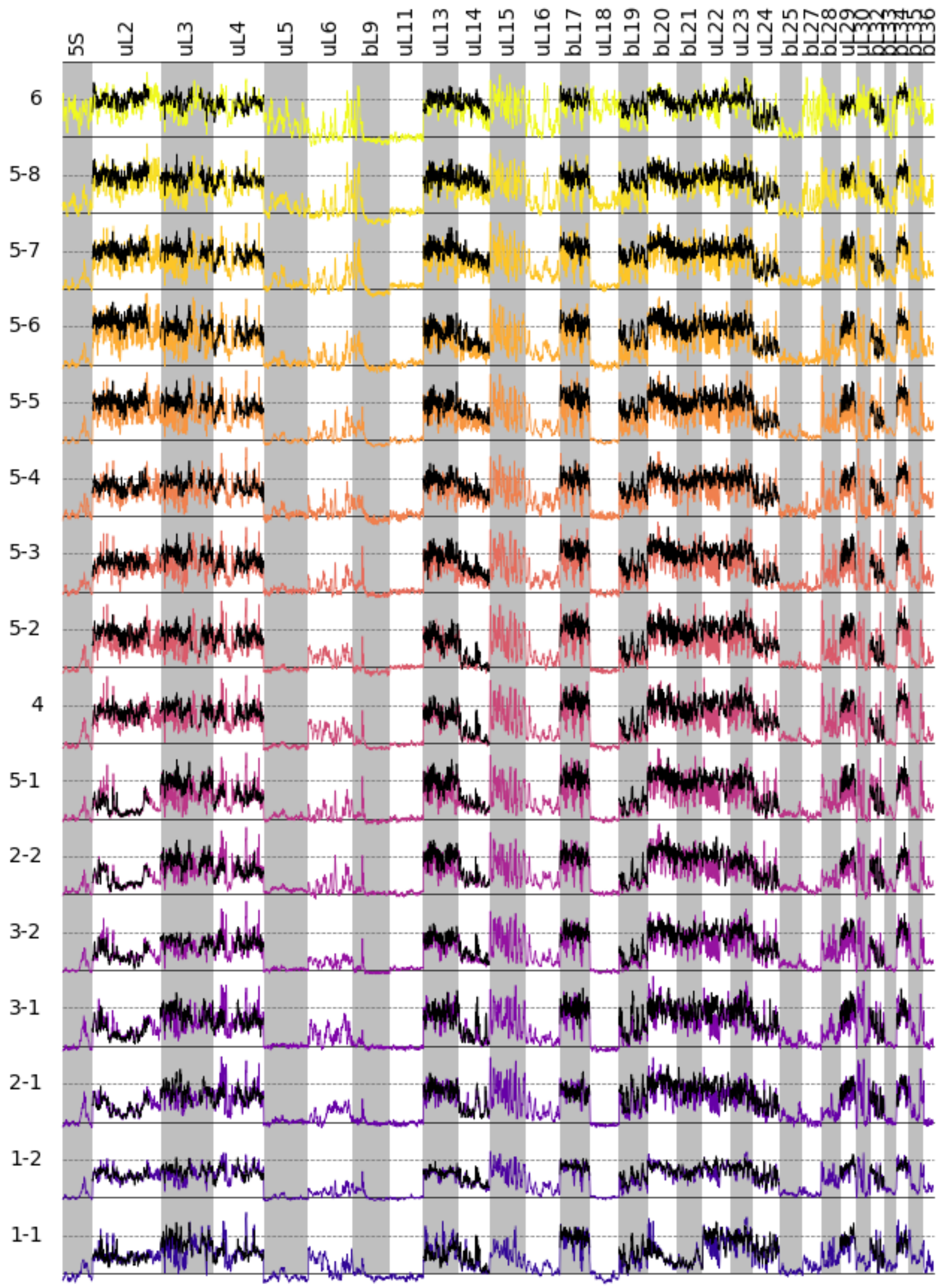

Figure S11. Correlation between map and model for 5S rRNA and r-proteins in the LSU as a function of residue number, calculated as the average map values at heavy atom positions for each nucleotide in rigid-body docked atomic models. Colored lines are for the mature ribosome (PDB ID 4YBB) and black lines are for a model refined against the consensus reconstruction. Vertical gray and white bars indicate the extension of chains. Dashed horizontal lines indicate a map value of 0.05 (threshold used for occupancy calculations in Figure S12).

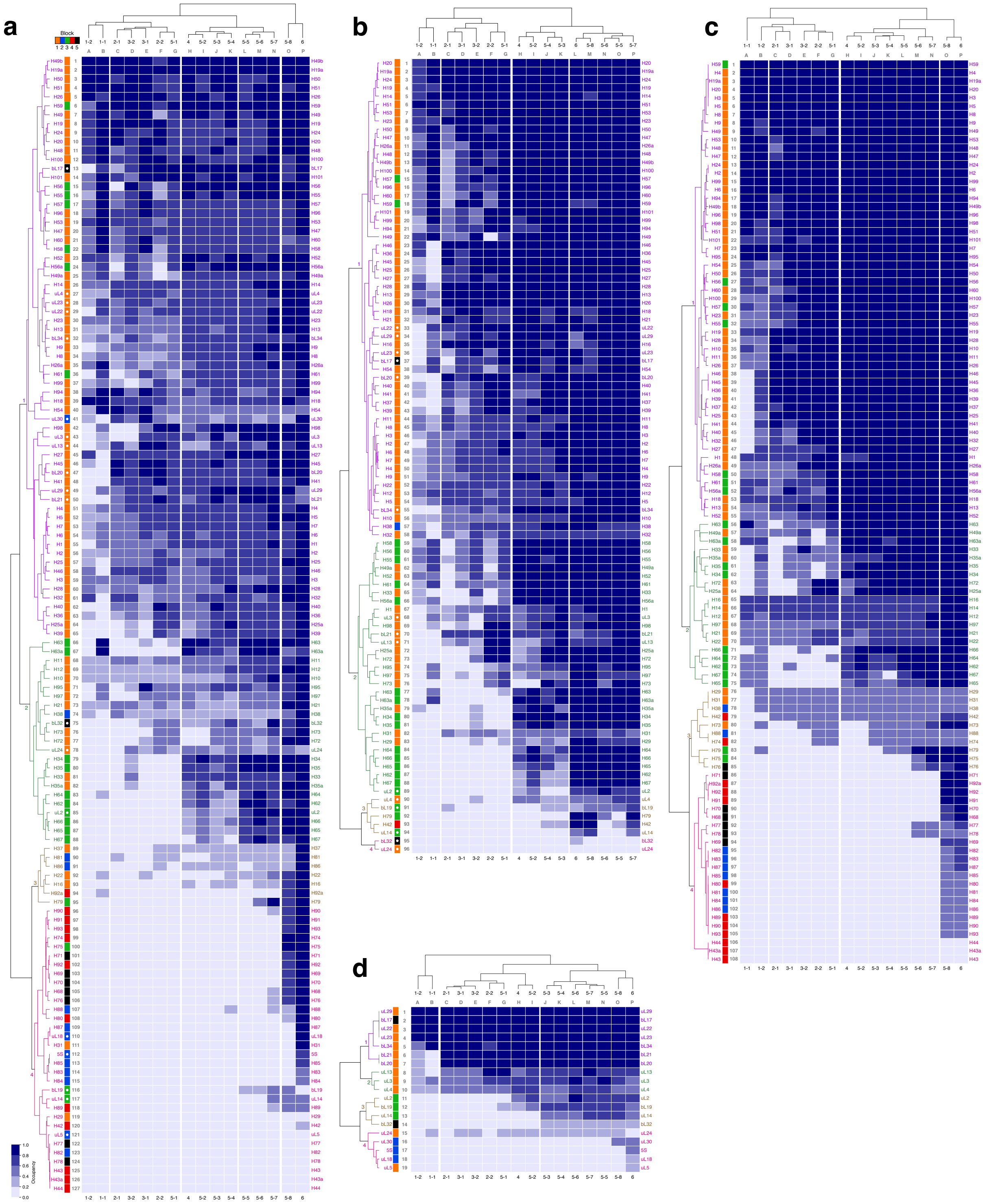
a
b
c
d
Block
Occupancy

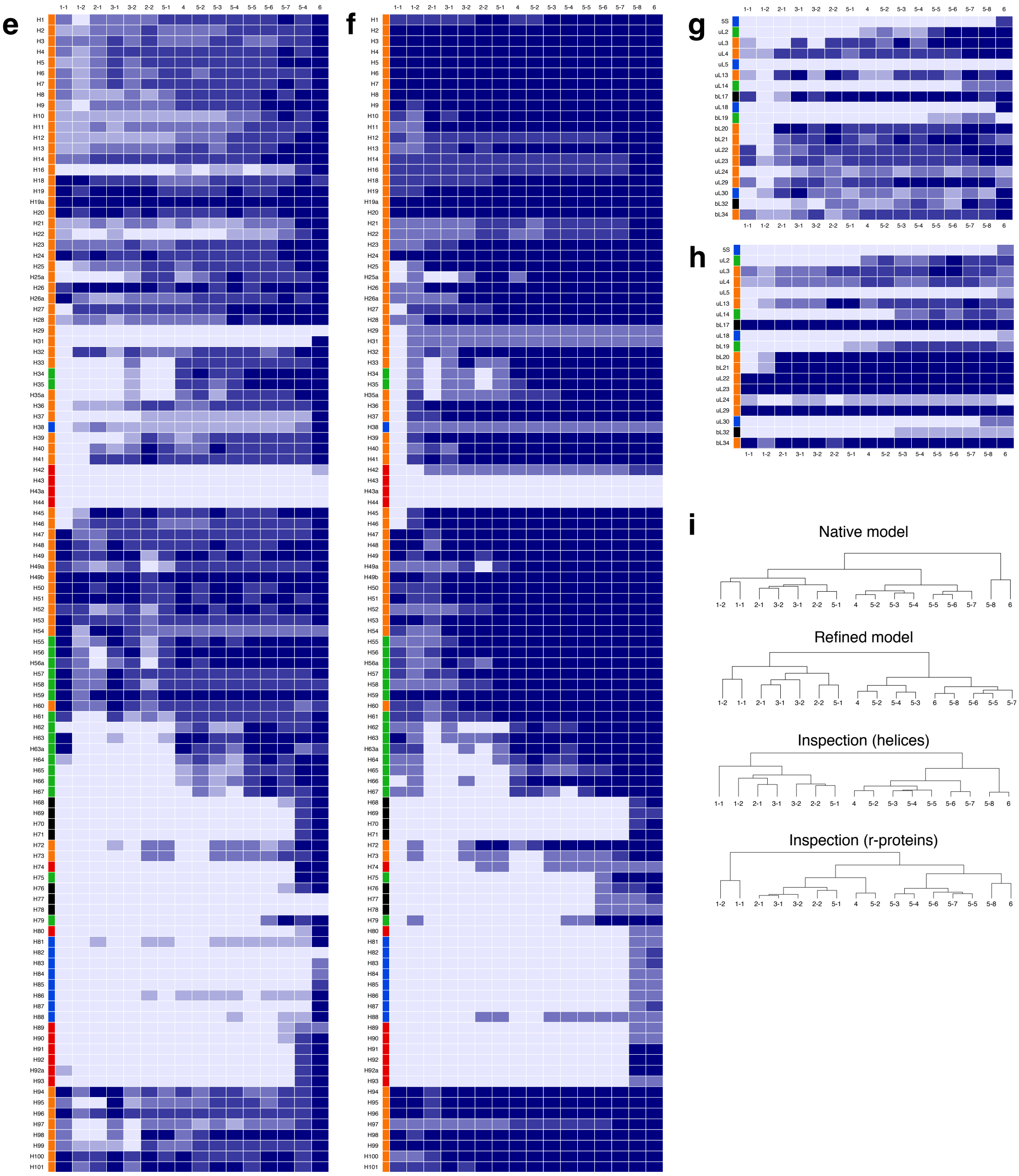

Figure S12. Occupancy and clustering of structural elements in the reconstruction classes. (**a**) The occupancy is estimated for each r-protein or 23S rRNA SSE as the map values at the heavy-atom positions for a rigid-body docked atomic model of the mature ribosome (PDB ID: 4YBB, *cf.* Figures S10 and S11) and normalized according to [3]. R-proteins not present in any of the classes were excluded. Dendrograms at the top and left show the clustering between columns and rows, respectively, according to complete linkage hierarchical clustering (see methods for details). Branches in the dendrograms are arranged to place higher average values closer to the top and to the right. The four deepest branches in the row dendrogram are numbered 1–4 and are colored differently to guide the eye. The color legend for rows indicate the folding block as described in [3] and r-proteins and 5S rRNA are identified with an additional white dot. Thick vertical lines in the heatmap indicate deep branches in the column dendrogram. (**b**) Equivalent to (a), but calculated for a model refined to the consensus reconstruction. (**c**) Occupancy estimated for 23S rRNA SSE by manual inspection of maps and then analyzed in the same way as in (a). Values were assigned based

on the appearance and position of the density. (**d**) Occupancy estimated for r-protein and 5S rRNA by manual inspection of maps and analyzed in the same way as in (a). Values were set to the highest map threshold where distinct features could be recognized. (**e–h**) Side-by-side comparisons between the heatmaps in (a, c and d), respectively, but with the same column and row orders. (**e**) RNA in (a) (**f**) same as (c) (**g**) Proteins in (a) (**h**) same as (d) (**i**) Side-by-side comparison of column dendrograms in (a-d).

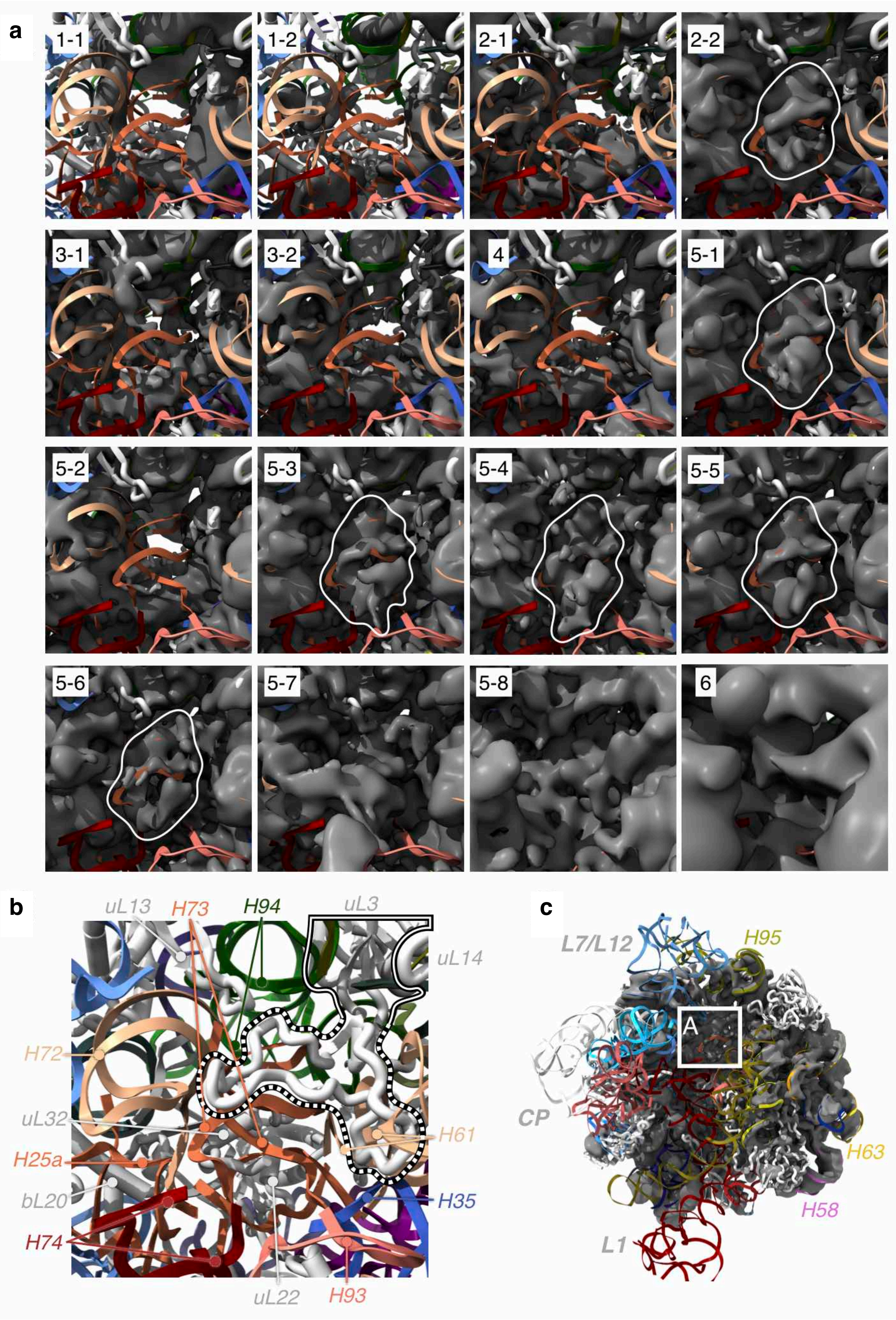
a
1-1
1-2
2-1
2-2
3-1
3-2
4
5-1
5-2
5-3
5-4
5-5
5-6
5-7
5-8
6
b
uL13
H73
H94
uL3
uL14
H72
uL32
H25a
bL20
H74
uL22
H93
H35
H61
c
L7/L12
H95
A
CP
H63
H58
L1

Figure S13. Related to Figure 4. Fold of helix H73. (**a**) Density for helix H73 (outlined in light gray) negatively correlate with presence of loop density (Figure 4a, star) (compare 2-1 vs 2-2, 4 vs 5-1 and 5-2 vs 5-3). H73 is positively correlated with two other domain-0 helices H25a and H72. Parts of model 4YBB are shown for clarity (disordered helices, such as H68-71 and H89–92a are not shown as well as disordered loop 128–162 of uL3). (**b**) Model 4YBB shown in the same way as in (a) without any density map, but with loop 128–162 of uL3 shown (dashed line). This loop interacts with H61, H72 and H73 of domain 0 as well as H94, H96 and H100 of domain VI. (**c**) Overview.

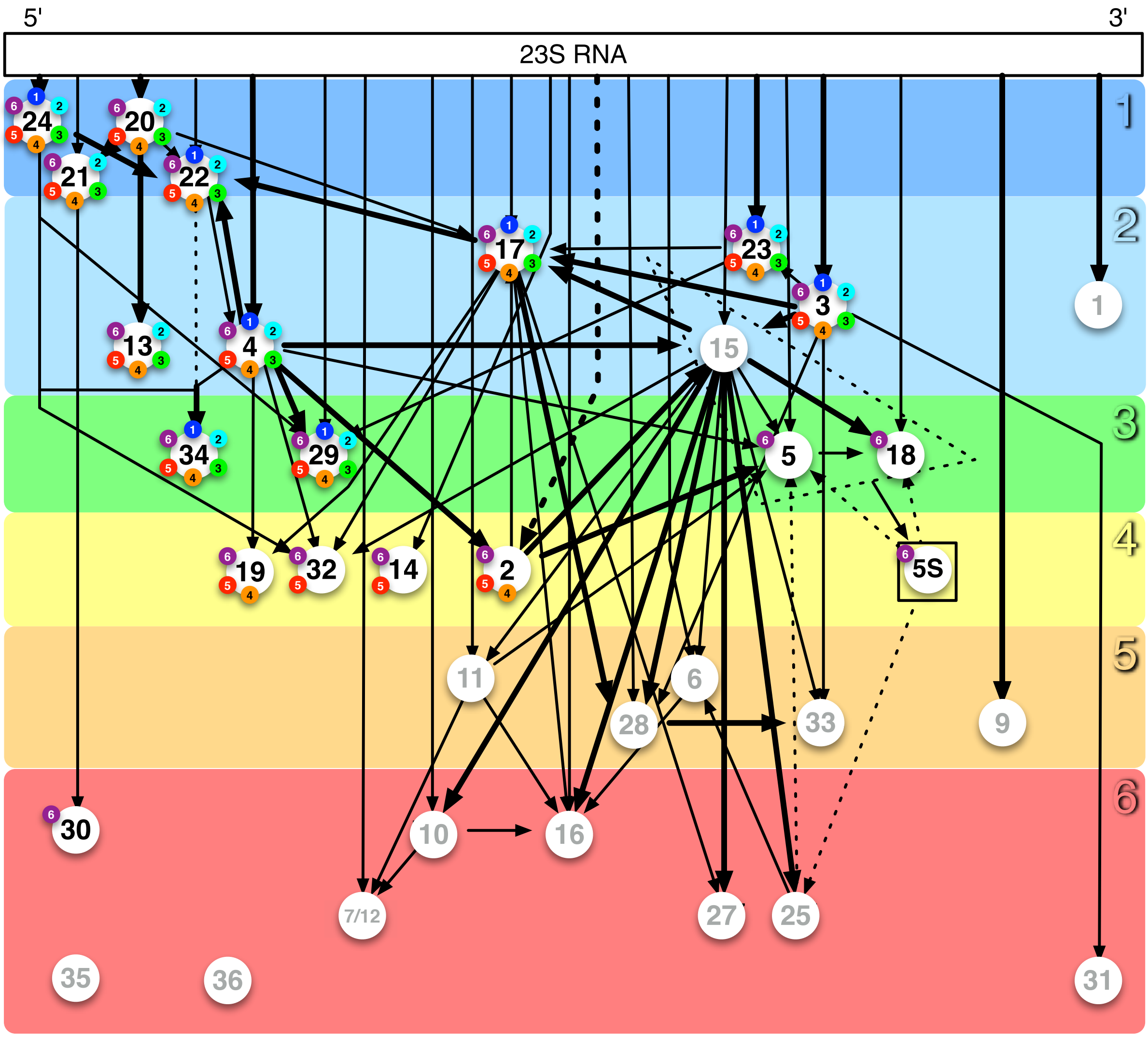

Figure S14. *In vivo* assembly groups (boxes 1–6, colored from the top: 1-dark blue, 2-light blue, 3-green, 4-yellow, 5-orange and 6-red) of r-proteins associated with the *E. coli* ribosomal LSU with arrows indicating interactions between proteins and to the 23S rRNA, as determined by [5]. R-proteins confirmed to be present in the cryo-EM reconstructions of LiCl core particles are indicated with black numbers (*cf.* Figure S11 and Table S4) and absent r-proteins with gray numbers. Small colored circles (1-blue, 2-cyan, 3-green, 4-orange, 5-red and 6-purple) show in which classes the proteins are observed. Some proteins are only present at reduced occupancy (*e.g.* uL24 and bL32) or only in some sub-classes. Adopted from [5] with permission.

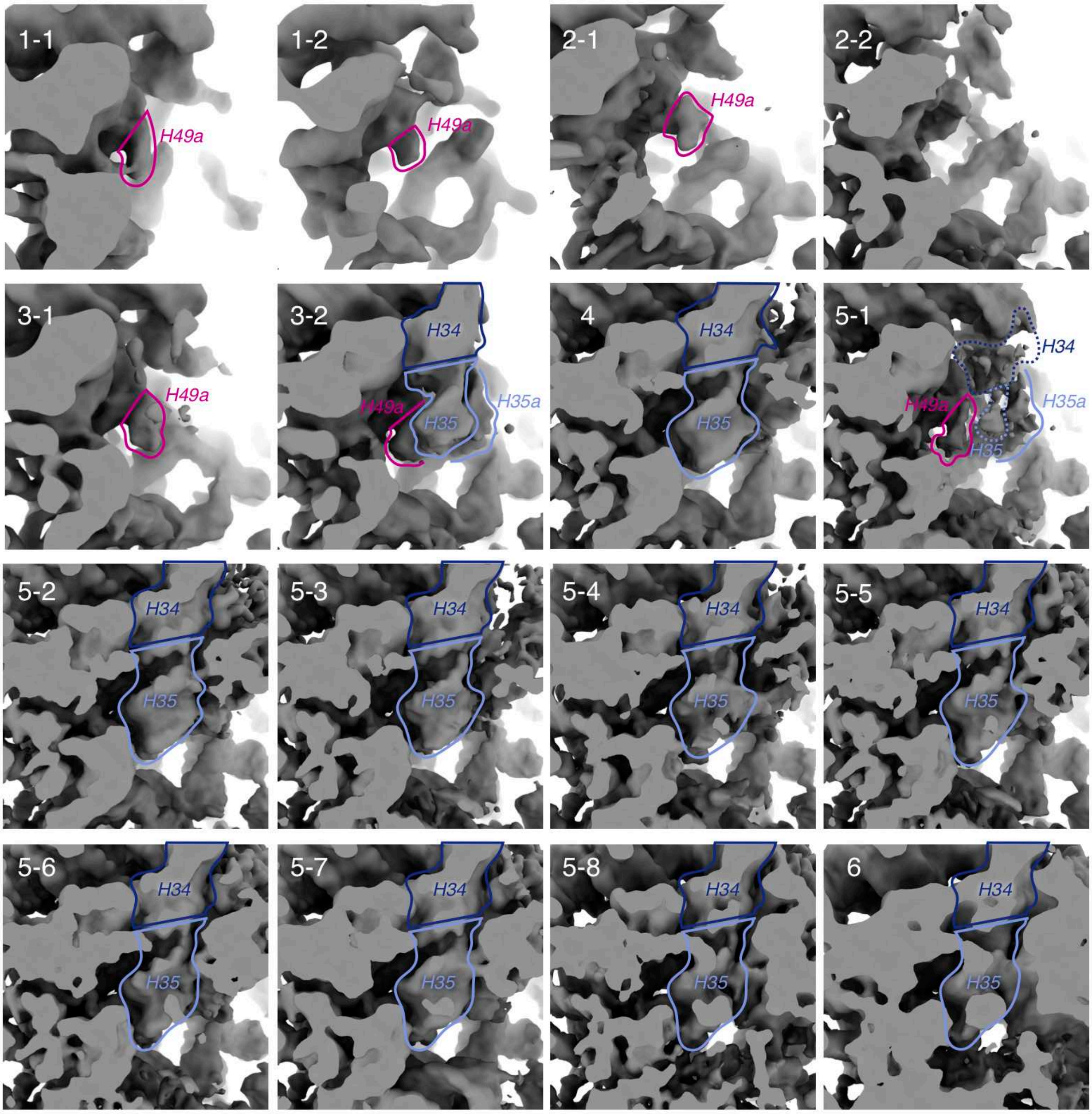

Figure S15. Related to Figure 7. Folding of the H49a-H35 region. Full gallery of classes rendered as in Figure 7a. Helices H49a and H34–35a are outlined.

Supplementary Tables

Table S1. Cryo-EM data collection, reconstruction and modelling statistics

**Data collection**	
Electron microscope	Titan Krios
Camera	Falcon 3 (linear mode)
Magnification	75,000
Pixel size at detector [Å]	1.07
Defocus range [µm]	-0.5 to -3.5
Voltage [kV]	300
Stage tilt	0° / 15° / 30°
Electron dose [e^-/Å^2] (0° / 15° / 30°)	41.0 / 41.0 / 42.2
Number of micrographs	11,368
- Per tilt (0° / 15° / 30°)	3,052 / 2,196 / 6,120
**Map refinement (consensus reconstruction)**	
Number of particles	384,374
- Per tilt (0° / 15° / 30°)	104,787 / 88,316 / 191,271
Map resolution, half-map $FSC_{0.143}$ [Å]	2.84
Map sharpening B-factor [Å^2]	-32.2
**Model statistics (consensus reconstruction)**	
Model composition	
Total atoms (all / non-hydrogen)	92,575/ 56,530
RNA residues	1,990
Protein residues	1,761
General	
Map-model $FSC_{0.5}$ [Å]	2.91
Map-model CC_{mask}	0.817
MolProbity score	1.49
Clash score	2.99
R.m.s. deviations bond length [Å]	0.0022
R.m.s. deviations bond angles [°]	0.36
Protein geometry	
Rotamer outliers [%]	3.32
Cβ deviations >0.25 Å [%]	0.00
Ramachandran [%] (Fav. / Allow. / Outl.)	98.97 / 2.03 / 0.00
Rama-Z (whole / helix / sheet / loop)	-0.12 / 0.41 / -0.50 / 0.02
CaBLAM [%] (Outl. / Disf.)	1.06 / 4.78
EMRinger score	3.43
Nucleic acid geometry	
Backbone bond length outliers [%]	0.007
Backbone bond angle outliers [%]	0.000
Sugar puckers outliers [%]	0.050
Backbone bond torsion suite outliers [%]	11.4%
Backbone average suiteness	0.636

Table S2. Details about cryo-EM reconstruction classes

Class	No. particles	$FSC_{0.143}$ [Å]	B-factor [$Å^2$]	E_{od}[1]	Vol. high[2] [$Å^3$]	Vol. low[2] [$Å^3$]	EMDB-ID	PDB-ID
consensus	384,374	2.84	-32.2	0.7	457,870	1,129,100	EMD-12826	7ODE
1-1	3,425	9.40	-100	0.7	383,810	1,160,000	EMD-12828	-
1-2	16,155	9.22	-300	0.8	238,820	1,376,100	EMD-12829	-
2-1	7,727	7.61	-150	0.7	435,630	1,207,300	EMD-12830	-
2-2	23,545	4.32	-68.1	0.7	456,270	1,148,500	EMD-12831	-
3-1	10,971	6.31	-110	0.7	507,910	1,212,200	EMD-12832	-
3-2	31,459	4.28	-70.0	0.8	445,430	1,087,400	EMD-12833	-
4	53,422	3.33	-41.8	0.7	494,250	1,135,100	EMD-12834	-
5-1	24,528	3.38	-26.0	0.8	465,970	1,111,800	EMD-12835	-
5-2	21,489	3.28	-16.7	0.7	505,730	1,106,000	EMD-12836	-
5-3	14,756	3.47	-25.4	0.6	542,250	1,219,300	EMD-12838	-
5-4	3,966	4.94	-50.5	0.6	567,120	1,338,200	EMD-12837	-
5-5	133,733	2.91	-23.8	0.6	497,210	1,150,400	EMD-12839	-
5-6	11,792	3.66	-33.9	0.6	598,290	1,269,100	EMD-12840	-
5-7	12,275	3.80	-29.3	0.7	623,400	1,382,400	EMD-12841	-
5-8	9,112	4.57	-48.1	0.5	728,520	1,593,100	EMD-12843	-
6	2,103	9.22	-63.8	0.6	839,210	1,982,500	EMD-12844	-

[1] Efficiency of Orientation Distribution, calculated by cryoEF [6]

[2] Volume inscribed by colored or semi-transparent surfaces with a high (0.10) or low (0.05) threshold, respectively, in Figure S6, calculated by the 'measure volume' command of UCSF Chimera [7].

Table S3. Naming of "non-standardized" 23S secondary structure elements

Nucleotides	Petrov *et al.* 2013[1]	This study	Davis *et al.* 2017[2]
319–320	no data	H19a	H19 (299–320)
321–323	no data	H19a	H20 (321–339)
562–578	H25a	H25a	H102
776–789	H35a	H35a	H103
1262–1270, 2010–2017	H26a	H26a	H104
1307–1313	H49b	H49b	H105
1565–1568	no data	H56a	H106
1603–1608	H49b	H49b	H107 (1603–1621)
1609–1621	H49a	H49a	H107 (1603–1621)
1752–1753	no data	H63a	H63 (1707–1753)
1754–1756	no data	H63a	H64 (1754–1773)
1925–1931	no data	H70	H70
2562–2566	no data	H92a	H91 (2518–2546, 2562–2566)

[1] [8], specifically the SSE diagram accessed on September 22, 2020 at http://apollo.chemistry.gatech.edu/RibosomeGallery/bacteria/E%20coli/LSU/E_coli_LSU_Helices_2.png

[2] [9], specifically the segmentation in Supplementary Table 3

Table S4. R-protein composition of LiCl core particles in literature, as supported by LC-MS/MS or cryo-EM data, and in comparison with early *in vitro* assembly precursors and bL17 depletion assembly intermediates. Brackets indicate reduced amounts or weak density.

	4.2 M core[1]	4.0 M core[2]	3.5 M core[3]	LC-MS/MS (Score)[4]	LC-MS/MS (PSM/Unique)[5]	Consensus	1-1	1-2	2-1	2-2	3-1	3-2	5-1	4	5-2	5-3	5-4	5-5	5-6	5-7	5-8	6	$RI_{50}(1)$[1]	S1[6]	S2[6]	B[7]	C[7]	D1[7]
**uL1**				59.2	2.60																							
**uL2**	(+)		(+)	360.6	7.00	(+)								+	+	+	+	+	+	+	+	+			+		+	
**uL3**	+	+	+	164.6	6.08	+	+	(+)	+	+	+	+	+	+	+	+	+	+	+	+	+	+		+	+	+	+	+
**uL4**	+	+	+	76.3	6.17	+	(+)	(+)	+	+	+	+	+	+	+	+	+	+	+	+	+	+	+	+	+	+	+	+
**uL5**				67.7	2.50																	(+)						+
**uL6**				8.6	1.25																				(+)			
**bL9**				33.4	2.00																							
**uL10**				ND	ND																							
**uL11**				9.4	1.25																							
**bL12**				ND	ND																							
**uL13**	+	+	+	126.3	6.67	+		(+)	+	+	+	+	+	+	+	+	+	+	+	+	+	+	+	+	+	+	+	+
**uL14**				53.1	5.17	(+)										+	+	+	+	+	+	+		+	+		+	
**uL15**				28.3	1.63																			+	+	+	+	+
**uL16**				ND	ND																							
**bL17**	(+)	(+)	+	132.5	9.14	+	+	+	+	+	+	+	+	+	+	+	+	+	+	+	+	+		+	+			
**uL18**				9.5	1.33																(+)	(+)						+
**bL19**				94.1	4.86	(+)							(+)	(+)	+	+	+	+	+	+	+	+		+	+		+	
**bL20**	+		+	94.2	5.89	+	(+)	(+)	+	+	+	+	+	+	+	+	+	+	+	+	+	+	+	+	+	+	+	+
**bL21**	(+)	(+)	+	72.8	5.83	+	(+)	(+)	+	+	+	+	+	+	+	+	+	+	+	+	+	+		+	+	+	+	+
**uL22**	(+)	+	+	140.9	5.00	+	+	+	+	+	+	+	+	+	+	+	+	+	+	+	+	+	+	+	+	+	+	+
**uL23**	(+)	(+)	+	24.8	3.00	+	+	+	+	+	+	+	+	+	+	+	+	+	+	+	+	+		+	+	+	+	+
**uL24**				61.7	3.33	(+)	(+)		(+)	(+)	(+)	(+)	(+)	(+)	(+)	(+)	(+)	(+)	(+)	(+)	(+)	(+)	+	+	+	+	+	+
**bL25**				ND	ND																							+
**bL27**				ND	ND																							+
**bL28**				ND	ND																							
**uL29**		(+)	+	37.4	5.33	+	+	+	+	+	+	+	+	+	+	+	+	+	+	+	+	+		+	+	+	+	+
**uL30**				11.1	1.75																+	+		+	+			+
**bL31**				7.5	1.33																					+	+	+
**bL32**		(+)		12.7	1.67	(+)										(+)	(+)	(+)	(+)	(+)	(+)	(+)		+	+			
**bL33**				ND	ND																							
**bL34**			+	ND	ND	+	+	+	+	+	+	+	+	+	+	+	+	+	+	+	+	+		+	+	+	+	+
**bL35**				ND	ND																							
**bL36**				ND	ND																							

[1] Ref. [10]
[2] Ref. [11]
[3] Ref. [12]
[4] Sequest algorithm protein score (Proteome Discoverer 1.4, Thermo Fisher Scientific)
[5] The total number of identified peptide sequences (peptide spectrum matches, PSM) for the protein divided by the number of unique peptide sequences to estimate the relative r-protein abundance.
[6] Ref. [4]
[7] Ref. [3]

Supplementary References

1. Zivanov, J.; Nakane, T.; Forsberg, B.O.; Kimanius, D.; Hagen, W.J.H.; Lindahl, E.; Scheres, S.H.W. New Tools for Automated High-Resolution Cryo-EM Structure Determination in RELION-3. *Elife* **2018**, *7*, e42166, doi:10.7554/eLife.42166.
2. Zhang, K. Gctf: Real-Time CTF Determination and Correction. *J. Struct. Biol.* **2016**, *193*, 1–12, doi:10.1016/j.jsb.2015.11.003.
3. Davis, J.H.; Tan, Y.Z.; Carragher, B.; Potter, C.S.; Lyumkis, D.; Williamson, J.R. Modular Assembly of the Bacterial Large Ribosomal Subunit. *Cell* **2016**, *167*, 1610–1622, doi:10.1016/j.cell.2016.11.020.
4. Nikolay, R.; Hilal, T.; Qin, B.; Mielke, T.; Bürger, J.; Loerke, J.; Textoris-Taube, K.; Nierhaus, K.H.; Spahn, C.M.T. Structural Visualization of the Formation and Activation of the 50S Ribosomal Subunit during In Vitro Reconstitution. *Mol. Cell* **2018**, *70*, 881–893, doi:10.1016/j.molcel.2018.05.003.
5. Chen, S.S.; Williamson, J.R. Characterization of the Ribosome Biogenesis Landscape in E. Coli Using Quantitative Mass Spectrometry. *J. Mol. Biol.* **2013**, *425*, 767–779, doi:10.1016/j.jmb.2012.11.040.
6. Naydenova, K.; Russo, C.J. Measuring the Effects of Particle Orientation to Improve the Efficiency of Electron Cryomicroscopy. *Nat. Commun.* **2017**, *8*, 629, doi:10.1038/s41467-017-00782-3.
7. Pettersen, E.F.; Goddard, T.D.; Huang, C.C.; Couch, G.S.; Greenblatt, D.M.; Meng, E.C.; Ferrin, T.E. UCSF Chimera - A Visualization System for Exploratory Research and Analysis. *J. Comput. Chem.* **2004**, *25*, 1605–1612, doi:10.1002/jcc.20084.
8. Petrov, A.S.; Bernier, C.R.; Hershkovits, E.; Xue, Y.; Waterbury, C.C.; Hsiao, C.; Stepanov, V.G.; Gaucher, E.A.; Grover, M.A.; Harvey, S.C.; et al. Secondary Structure and Domain Architecture of the 23S and 5S RRNAs. *Nucleic Acids Res.* **2013**, *41*, 7522–7535, doi:10.1093/nar/gkt513.
9. Davis, J.H.; Williamson, J.R. Structure and Dynamics of Bacterial Ribosome Biogenesis. *Philos. Trans. R. Soc. B Biol. Sci.* **2017**, *372*, 20160181, doi:10.1098/rstb.2016.0181.
10. Spillmann, S.; Dohme, F.; Nierhaus, K.H. Assembly in Vitro of the 50 S Subunit from Escherichia Coli Ribosomes: Proteins Essential for the First Heat-Dependent

Conformational Change. *J. Mol. Biol.* **1977**, *115*, 513–523, doi:10.1016/0022-2836(77)90168-1.

11. Homann, H.E.; Nierhaus, K.H. Ribosomal Proteins: Protein Compositions of Biosynthetic Precursors and Artifical Subparticles from Ribosomal Subunits in Escherichia Coli K 12. *Eur. J. Biochem.* **1971**, *20*, 249–257, doi:10.1111/j.1432-1033.1971.tb01388.x.
12. Nierhaus, K. Reconstitution of Ribosomes. In *Ribosomes and Protein Synthesis*; Spedding, G., Ed.; Oxford University Press: Oxford, 1990; pp. 161–189.
